# Cross-regional comparison of university teachers’ intentions to adopt digital teaching in China: a multi-group SEM analysis based on UTAUT

**DOI:** 10.3389/fpsyg.2026.1899398

**Published:** 2026-08-11

**Authors:** Yu-Hang Ma, Jie Yang

**Affiliations:** Faculty of Education, Shaanxi Normal University, Xi'an, China

**Keywords:** digital teaching, multi-group structural equation modeling, regional differences, teacher adoption intention, UTAUT

## Abstract

The Unified Theory of Acceptance and Use of Technology (UTAUT) is a dominant framework for explaining teachers’ technology acceptance, yet its assumption of cross-contextual stability has rarely been challenged. Region, as a composite variable carrying multiple structural differences such as resource adequacy and professional community density, may systematically moderate the strength of UTAUT paths. This study surveyed 351 university teachers from eastern (*n* = 126), central (*n* = 115), and western (*n* = 110) China and employed multi-group structural equation modeling to examine cross-regional differences in the core paths of UTAUT. The results showed that at the full-sample level, performance expectancy (*β* = 0.32), effort expectancy (*β* = 0.14), social influence (*β* = 0.21), and facilitating conditions (*β* = 0.26) all significantly and positively predicted adoption intention, jointly explaining 51.2% of the variance. At the cross-regional level, effort expectancy explained adoption intention more strongly for western than for eastern teachers (*β*_west = 0.23 vs. *β*_east = 0.09, *p* = 0.004); facilitating conditions showed a similar pattern (*β*_west = 0.34 vs. *β*_east = 0.21, *p* = 0.009); social influence explained adoption intention more strongly for eastern than for western teachers (*β*_east = 0.28 vs. *β*_west = 0.15, *p* = 0.003); and performance expectancy remained stable across the three regions (*β*_east = 0.31, *β*_central = 0.33, *β*_west = 0.29, *p* = 0.654). These findings suggest that the path weights of UTAUT systematically vary with regional development levels. Accordingly, eastern universities should prioritize culture cultivation, western universities resource assurance, and central universities a dual-track strategy.

## 1 Introduction

### 1.1 From technological availability to teacher adoption: a persistent translation gap

Higher education worldwide is undergoing a systemic transformation from traditional face-to-face instruction to blended and online teaching (Martin et al., 2009). Digital teaching, defined as the systematic integration of learning management systems, interactive tools, and intelligent teaching platforms into instructional activities, has been shown to enhance student engagement, facilitate personalized learning, and strengthen pedagogical resilience (Clark-Wilson et al., 2020; Basilotta-Gómez-Pablos et al., 2022; ElSayary, 2024). However, a recurring and puzzling phenomenon for policymakers is that substantial investments in technological infrastructure have not automatically translated into meaningful changes in teachers’ daily instructional practices. Hardware has been installed, platforms launched, and training conducted. Yet a considerable number of teachers still use digital technologies as presentation tools rather than as pedagogical norms. This gap suggests that technological availability alone does not sufficiently drive adoption. Instead, teachers’ adoption intention constitutes the core psychological mechanism for bridging the last mile (Claro et al., 2018).

### 1.2 Strengths and blind spots of UTAUT

In the theoretical landscape explaining teachers’ technology adoption intentions, the Unified Theory of Acceptance and Use of Technology (UTAUT) has become the most widely adopted analytical framework. Its popularity stems from its dual focus on individual cognitive factors, namely performance expectancy and effort expectancy, as well as contextual support factors, namely social influence and facilitating conditions (Yee and Abdullah, 2021; Wijaya et al., 2022; Wong et al., 2013). Compared with earlier technology acceptance models (TAM) (Scherer et al., 2019; Weng et al., 2018), UTAUT makes a core contribution by recognizing that adoption behavior can be hindered by a lack of organizational support and technological assurance, even when individual intentions are fully present. This feature renders UTAUT particularly suitable for the organizational context of higher education.

However, the very prevalence of UTAUT has exposed a deeper problem. When a large body of studies repeatedly validates the same significant paths across different samples, researchers may have overlooked the contextual boundaries within which these paths hold. A close examination of the existing literature reveals that the vast majority of UTAUT studies operationalize context as individual level moderators, such as gender, age, experience, and voluntariness, while rarely examining how macro structural factors systematically shape the formation mechanisms of adoption intention. This tendency has led to a theoretical blind spot. UTAUT is treated as a context free universal model, as if its path coefficients were stable across any organizational environment.

### 1.3 The overlooked structural variable: region

In research on digital teaching in higher education, one particularly important yet persistently marginalized macro level variable is region. Region is not a simple geographical label. Instead, it is a composite variable carrying multiple structural differences (Hu, 2003). Significant gradients exist across regions in terms of economic development levels, educational funding, informatization progress, and the allocation of higher education resources. These macro level structural disparities directly shape substantive differences in the digital teaching contexts faced by university faculty. These differences include the technological conditions available to teachers, the training support they can access, and the digital teaching culture surrounding them.

This implies that the digital teaching contexts faced by university teachers in eastern, central, and western China are fundamentally different. An adoption intention model validated on an eastern sample may not have path coefficients directly applicable to teachers in central or western regions. Consequently, a critical theoretical question emerges. Do the influence paths of UTAUT have the same strength across different regional groups of teachers?

Regrettably, existing studies either draw inferences based on single region samples, for instance by surveying only universities in one province, or statistically control for region as a demographic variable. Very few have systematically employed region as a core comparative dimension using multi group analysis. This gap not only results in a theoretical contextual blind spot but also directly leads to one size fits all policy recommendations. Such recommendations lack empirical justification yet remain widely observed in practice.

### 1.4 The present study’s choices

Based on the above analysis, this study makes two key decisions.

First, we exclude UTAUT’s original moderators. This study does not incorporate gender, age, experience, or voluntariness as moderating variables for the following reasons. Among university faculty, age and experience are heavily concentrated in the mid career stage with limited variance. The moderating effects of gender in digital teaching adoption research have consistently been unstable. Within China’s institutional environment, digital teaching has gradually shifted from voluntary exploration to institutional expectation, rendering voluntariness significantly diminished in explanatory power. As will be detailed in the sample description in Section 3.1, the limited variance in age and experience within this sample, combined with the absence of significant gender effects in preliminary analyses, justifies excluding these moderators. Retaining them would introduce unnecessary noise and dilute the focus on our core research question.

Second, we establish region as the core comparative dimension. Rather than treating region as a moderator within the model, that is constructing interaction terms, we adopt multi group structural equation modeling as our analytical framework. This methodological choice has clear justifications. When the moderator is a categorical variable with more than two categories, such as eastern, central, and western, multi group comparison is statistically cleaner. It does not require the introduction of multiple dummy variables and their product terms, thereby avoiding the multicollinearity and estimation instability often associated with complex interaction models (Aiken and West, 1991). It is also more intuitive in interpretation, allowing researchers to directly present path coefficients for each group and conduct pairwise comparisons. More importantly, multi group analysis permits tests of measurement invariance, a prerequisite for valid cross regional comparisons that traditional moderation analysis cannot provide.

### 1.5 Research objectives

In summary, this study aims to answer two core research questions.

Research Question 1: Within the core path model of UTAUT, do performance expectancy, effort expectancy, social influence, and facilitating conditions significantly affect university teachers’ intentions to adopt digital teaching?

Research Question 2: Do the above path coefficients differ significantly among eastern, central, and western teacher groups? If so, what is the specific pattern of these differences?

By answering these questions, this study seeks to provide empirical evidence from Chinese higher education regarding the contextual boundaries of UTAUT at the theoretical level. At the practical level, it aims to offer targeted decision making bases for differentiated digital teaching promotion strategies across regional university contexts.

## 2 Research hypotheses

### 2.1 Core path hypotheses of UTAUT

Performance expectancy refers to the degree to which a teacher perceives that using digital teaching can enhance their teaching effectiveness. In the higher education context, performance expectancy encompasses several specific aspects, including increasing student classroom engagement, optimizing the traceability of teaching evaluation data, enabling more precise learning diagnostics, and innovating course design formats (Shah et al., 2021; Liu et al., 2025). Teachers, as bounded rational actors, allocate their time and energy to instructional activities with the highest expected returns. When teachers are convinced that digital teaching can bring observable pedagogical gains, their adoption intention should increase accordingly. This relationship has been repeatedly validated in research on teacher technology adoption, and its theoretical logic holds equally in the digital teaching context (Yahaya et al., 2022). Therefore, we propose:

*H1*: Performance expectancy has a significant positive effect on university teachers’ intentions to adopt digital teaching.

Effort expectancy refers to the perceived cognitive and operational costs that a teacher associates with using digital teaching. For digital teaching, the core concern of effort expectancy is how much additional time teachers need to invest in learning new tools, designing online activities, or troubleshooting technical problems. Cognitive load theory suggests that when the demands of a technology exceed an individual’s cognitive resource capacity, the individual tends to avoid using that technology (Pan and He, 2024; Al-Adwan et al., 2025). Although some argue that the influence of effort expectancy diminishes once teachers overcome the initial skill threshold, most university teachers remain in a state of perpetual learning given the continuous iteration of digital teaching technologies. For those who have not yet formed stable technology habits, ease of use remains an antecedent condition affecting adoption intention (Tang et al., 2024). Therefore, we propose:

*H2*: Effort expectancy has a significant positive effect on university teachers’ intentions to adopt digital teaching.

Social influence refers to the perceived pressure or expectation from important referents, such as colleagues, teaching deans, students, or academic affairs departments, regarding a teacher’s use of digital teaching. The mechanism of social influence can be distinguished into two types. The first is internalization, where teachers adopt digital teaching because they identify with the values of their reference group. The second is compliance, where teachers express adoption intention to meet external expectations. In the university organizational environment, both mechanisms operate simultaneously. On one hand, the instructional reform atmosphere within departments and the demonstration effects of exemplary cases shape professional norms. On the other hand, implicit technology expectations embedded in course evaluations, teaching competitions, or student ratings generate institutional pressure (Plageras et al., 2023; Guillén-Gámez et al., 2024; Yurdakul et al., 2014). Therefore, we propose:

*H3*: Social influence has a significant positive effect on university teachers’ intentions to adopt digital teaching.

Facilitating conditions refer to the degree to which a teacher perceives organizational and technological support for using digital teaching. This is the core variable distinguishing UTAUT from TAM, and its theoretical value lies in recognizing that adoption behavior can be hindered by a lack of adequate technical support, hardware guarantees, or institutional incentives, even when individual intentions are fully present. Specific manifestations of facilitating conditions include stable network and equipment access, accessible training programs, responsive technical support teams, and reasonable assessment incentive policies (Raja Yusof et al., 2017; Hu et al., 2020; Nikolic et al., 2024). For teachers, the perception of facilitating conditions is not merely an operational matter of whether they can use digital teaching. It also serves as a signal of whether the institution values digital teaching, and this signaling function itself shapes attitudes. Therefore, we propose:

*H4*: Facilitating conditions have a significant positive effect on university teachers’ intentions to adopt digital teaching.

### 2.2 Cross-regional difference hypotheses

Do the above four paths have the same strength among university teacher groups in eastern, central, and western China? The following section derives cross-regional hypotheses for each path. It should be noted that this study does not pre-specify a direction for H5, but proposes directional theoretical predictions for H6, H7, and H8.

#### 2.2.1 Regional differences in the performance expectancy path (H5)

The strength of the performance expectancy effect depends on teachers’ reference standards for good teaching and their judgments of possibility under resource constraints. In eastern universities, teachers have more opportunities to access high-level digital teaching cases, their reference points are higher, and their expectations for digital teaching to improve effectiveness are more specific and cautious. In central and western universities, if resource limitations prevent teachers from achieving ideal forms of digital teaching, the marginal effect of performance expectancy may be weakened by the gap between ideals and reality. However, an alternative possibility exists. In environments with stronger resource constraints, teachers may be more sensitive to any tool that can enhance teaching effectiveness. Because both logics coexist and direct prior evidence is lacking, this study does not propose a directional hypothesis.

*H5*: The path coefficient from performance expectancy to adoption intention differs significantly across regions.

#### 2.2.2 Regional differences in the effort expectancy path (H6)

The strength of the effort expectancy effect should depend inversely on the starting level of digital skills and the level of training support in teachers’ environments. This reasoning is based on the logic of skill internalization. When a skill is practiced repeatedly and becomes automated, operators no longer consciously perceive its difficulty, and this factor no longer becomes a significant consideration in decision making. In eastern universities, teachers typically experience more systematic and continuous digital training, have higher frequencies of technology contact in their daily teaching environments, and have partially internalized digital skills as part of their teaching routines. Under these conditions, the marginal explanatory power of effort expectancy is relatively low. Conversely, in western universities, if teachers still face basic technological operational thresholds, such as logging into platforms, uploading resources, or using interactive tools, then effort expectancy will constitute a substantive constraint on adoption intention. Therefore, we propose:

*H6*: The path coefficient from effort expectancy to adoption intention is significantly higher in western regions than in eastern regions.

#### 2.2.3 Regional differences in the social influence path (H7)

The operation of social influence depends on two conditions: the existence and visibility of reference others, and the relevance of the information conveyed by reference others to individual decision making. In denser professional communities, teachers’ decisions are more likely to be guided by peer behavior, a core insight of social learning theory. Eastern universities have denser academic communities, higher frequencies of instructional reform activities, and teachers more easily observe peer demonstration behaviors and perceive institutional expectations. In this environment, social influence is more likely to translate into individual adoption intention. In central and western universities, if digital teaching has not yet formed a widespread normative pressure, or if the behavior of reference others lacks informational value, such as when peers are also in a wait and see mode, the effect of social influence should be weaker. Therefore, we propose:

*H7*: The path coefficient from social influence to adoption intention is significantly higher in eastern regions than in central and western regions.

#### 2.2.4 Regional differences in the facilitating conditions path (H8)

The marginal effect of facilitating conditions on adoption intention should follow the law of diminishing marginal returns. As conditions themselves improve, the incremental effect of further improvement on intention diminishes. In resource rich environments, facilitating conditions have entered a saturation zone, where additional equipment upgrades or training increases have limited effects on teacher intention, and other factors such as performance expectancy and social influence become the primary drivers. In resource poor environments, facilitating conditions are the determining factor in whether teachers can take the first step. Without basic equipment and technical support, intention cannot translate into behavior, and intention itself may hardly form. Based on this logic, facilitating conditions should have stronger explanatory power for adoption intention among western teachers. Therefore, we propose:

*H8*: The path coefficient from facilitating conditions to adoption intention is significantly higher in western regions than in eastern regions.

**Figure 1:**
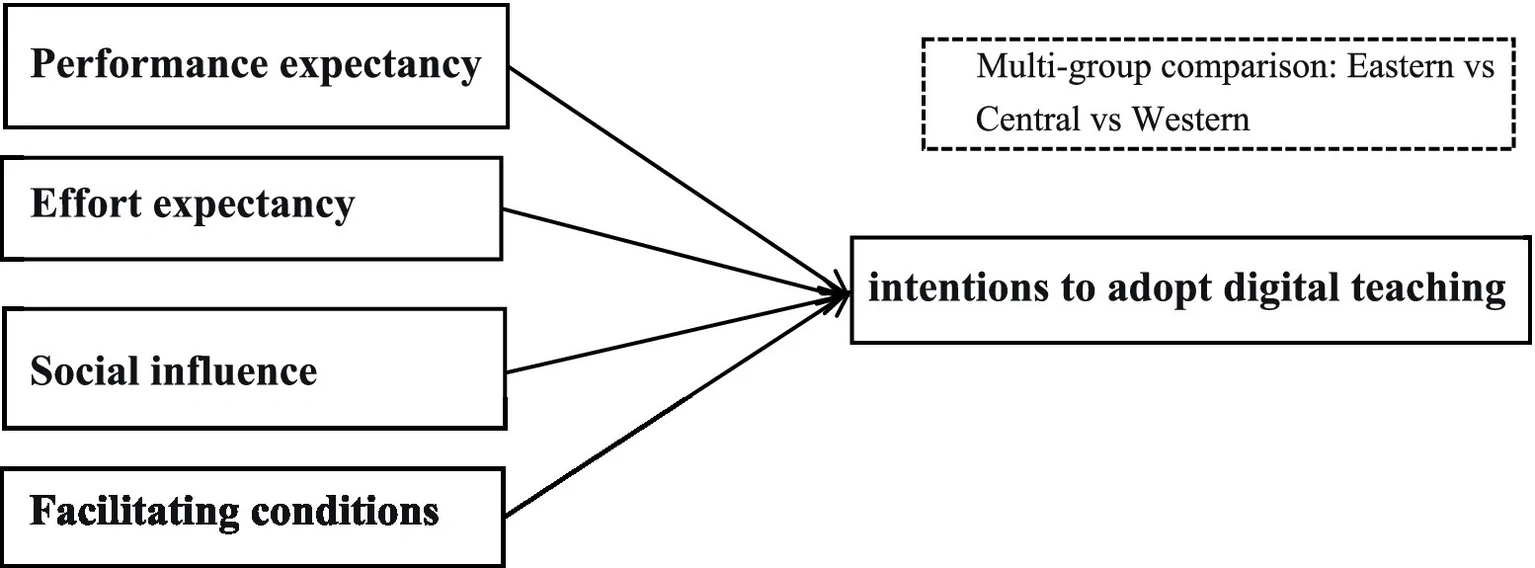
Theoretical model.

The theoretical model is presented in Figure 1.

## 3 Research methods

### 3.1 Sample and data collection

This study employed a stratified convenience sampling method to recruit full time teaching faculty from universities in eastern, central, and western China. Regional classification strictly followed the three major economic zones standard of the National Bureau of Statistics of China. The eastern region covers 11 provinces and municipalities, including Beijing, Shanghai, Jiangsu, Zhejiang, and Guangdong. The central region covers eight provinces, including Henan, Hubei, Hunan, and Anhui. The western region covers 12 provinces, autonomous regions, and municipalities, including Sichuan, Shaanxi, Chongqing, Yunnan, and Gansu. Stratified sampling was conducted within each region to ensure representativeness across different institutional levels.

Data collection was conducted through an online survey platform (Sojump). Invitations were distributed to teachers through the channels of university academic affairs offices and faculty development centers. The survey was administered over 12 weeks from September to December 2024. On the first page of the questionnaire, the research team clearly stated the purpose of the study, the principle of anonymity, and the commitment that data would be used solely for academic research. Participants were also informed of their right to withdraw at any time. All responses were considered as expressions of informed consent.

A total of 412 questionnaires were returned. The following exclusion criteria were applied: completion time of less than 3 min, indicating careless responding; obvious patterned response patterns, such as selecting the same option for all items; and missing values exceeding 10% on key variables. After data cleaning, 351 valid questionnaires were retained, yielding an effective response rate of 85.2%. The regional distribution of the sample was as follows: 126 cases (35.9%) from the eastern region, 115 cases (32.8%) from the central region, and 110 cases (31.3%) from the western region. All three group sample sizes met the recommended minimum of 100 cases per group for multi group structural equation modeling (Kline, 2023). Regarding sample characteristics, male teachers accounted for 46.7% and female teachers for 53.3%. The mean age was 38.9 years (SD = 9.5), and the mean teaching experience was 13.1 years (SD = 8.7). The professional title distribution was 11.7% full professors, 30.4% associate professors, 41.2% lecturers, and 16.8% assistant professors. The disciplinary distribution was 43.6% science and engineering, 41.5% humanities and social sciences, and 14.9% others. Cross-regionally, the proportions of science and engineering faculty were 44.4% in the east, 42.6% in the central region, and 43.6% in the west, with no significant differences across groups (*χ*^2^ = 4.12, df = 4, *p* = 0.390). Chi square tests and analysis of variance results showed no significant differences among the three groups in gender, age, teaching experience, or professional title distribution (*p* > 0.05), indicating that the three groups were comparable in basic demographic characteristics.

To further justify the exclusion of UTAUT’s original moderators, we examined the sample distribution of these variables. The standard deviations for age (SD = 9.5) and teaching experience (SD = 8.7) indicate that both variables were concentrated in the mid-career range, with limited representation at the extremes. Independent samples t-tests revealed no significant gender differences in adoption intention (*t* = 0.67, *p* = 0.503), and the correlation between age and adoption intention was weak (*r* = 0.13, *p* < 0.05), accounting for less than 2% of the variance. These sample characteristics support our decision to exclude these moderators, as their restricted range and weak associations with the dependent variable would yield unstable estimates if included.

### 3.2 Measurement instruments

All measurement items in this study were adapted from empirically validated scales in existing literature and were worded to fit the higher education digital teaching context. All constructs were measured on a 7 point Likert scale ranging from 1 (strongly disagree) to 7 (strongly agree), with higher scores indicating higher levels of agreement with the construct.

Performance expectancy was adapted from the teacher technology adoption scales of Venkatesh et al. (2003) and Scherer et al. (2019) to measure the degree to which a teacher perceives that using digital teaching can enhance their teaching effectiveness. The scale consisted of four items. Sample items include Using digital teaching tools can improve my teaching efficiency and Using digital teaching tools can improve student learning outcomes. In this study, the scale achieved a Cronbach’s alpha of 0.89, composite reliability (CR) of 0.91, and average variance extracted (AVE) of 0.72.

Effort expectancy was adapted from Venkatesh et al. (2003) to measure the perceived cognitive and operational costs associated with using digital teaching. The scale consisted of four items. Sample items include Learning to use digital teaching tools is easy for me and I can skillfully operate commonly used digital teaching platforms and tools. In this study, the scale achieved a Cronbach’s alpha of 0.87, CR of 0.89, and AVE of 0.68.

Social influence was adapted from Venkatesh et al. (2003) and Thompson et al. (1991) to measure teachers’ perceived expectations regarding digital teaching from colleagues, leaders, and the institutional environment. The scale consisted of four items. Sample items include Colleagues in my department generally believe that teachers should use digital teaching and University leaders support and encourage teachers to use digital teaching. In this study, the scale achieved a Cronbach’s alpha of 0.85, CR of 0.87, and AVE of 0.63.

Facilitating conditions were adapted from Venkatesh et al. (2003) to measure teachers’ perceived institutional support in terms of equipment, platforms, training, and technical assistance. The scale consisted of four items. Sample items include my university provides adequate digital teaching equipment, such as computers, internet access, and smart classrooms and my university provides accessible digital teaching platforms and technical support services. In this study, the scale achieved a Cronbach’s alpha of 0.88, CR of 0.90, and AVE of 0.70.

Digital teaching adoption intention was adapted from Venkatesh et al. (2003) and Davis (1989) to measure teachers’ subjective likelihood of actively using digital teaching tools in their future instruction. The scale consisted of three items. Sample items include I am willing to actively use digital teaching tools in my future teaching and If conditions permit, I will try digital teaching in more courses. In this study, the scale achieved a Cronbach’s alpha of 0.91, CR of 0.92, and AVE of 0.80.

Control variables included gender (1 = male, 2 = female), age (continuous variable), teaching experience (continuous variable), professional title (1 = assistant professor, 2 = lecturer, 3 = associate professor, 4 = full professor), and discipline category (1 = science and engineering, 2 = humanities and social sciences, 3 = others). These variables were included as covariates in subsequent multi group analysis to rule out potential confounding effects of demographic characteristics on the results.

All measurement items along with their factor loadings and reliability indicators are summarized in Table 1. Confirmatory factor analysis results showed that standardized factor loadings for all items ranged from 0.77 to 0.90 (*p* < 0.001). Composite reliability for each construct ranged from 0.87 to 0.92, and average variance extracted ranged from 0.63 to 0.80. All values exceeded the recommended thresholds of CR > 0.70 and AVE > 0.50 proposed by Fornell and Larcker (1981), indicating that the measurement model had good convergent validity.

**Table 1:** Summary of measurement items, factor loadings, and reliability.

Construct	Item code	Item content	Factor loading	Cronbach’s *α*	CR	AVE
Performance expectancy				0.89	0.91	0.72
	PE1	Using digital teaching tools can improve my teaching efficiency	0.83			
	PE2	Using digital teaching tools can improve student learning outcomes	0.87			
	PE3	Using digital teaching tools can help me better achieve teaching goals	0.85			
	PE4	Overall, I find digital teaching useful for my teaching work	0.86			
Effort expectancy				0.87	0.89	0.68
	EE1	Learning to use digital teaching tools is easy for me	0.81			
	EE2	I can skillfully operate commonly used digital teaching platforms and tools	0.84			
	EE3	Using digital teaching tools for instructional interaction is clear and easy to understand	0.79			
	EE4	Overall, using digital teaching tools does not require too much effort from me	0.85			
Social influence				0.85	0.87	0.63
	SI1	Colleagues in my department generally believe that teachers should use digital teaching	0.77			
	SI2	University leaders support and encourage teachers to use digital teaching	0.82			
	SI3	My teaching peers’ use of digital teaching has a positive influence on me	0.80			
	SI4	Overall, people around me think I should use digital teaching	0.78			
Facilitating conditions				0.88	0.90	0.70
	FC1	My university provides adequate digital teaching equipment (e.g., computers, internet, smart classrooms)	0.84			
	FC2	My university provides accessible digital teaching platforms and technical support services	0.86			
	FC3	My university has systematic digital teaching training programs for teachers	0.81			
	FC4	Overall, my university provides sufficient conditions and support for digital teaching	0.85			
Adoption intention				0.91	0.92	0.80
	BI1	I am willing to actively use digital teaching tools in my future teaching	0.89			
	BI2	I would recommend digital teaching methods to my colleagues	0.90			
	BI3	If conditions permit, I will try digital teaching in more courses	0.89			

Factor loadings are standardized coefficients, all significant at *p* < 0.001. CR = composite reliability. AVE = average variance extracted. All items were measured on a 7-point Likert scale ranging from 1 (strongly disagree) to 7 (strongly agree).

### 3.3 Data analysis strategy

This study adopted a two stage analytical strategy. In the first stage, IBM SPSS 26.0 was used to conduct descriptive statistical analysis, independent samples t tests, and one way analysis of variance to preliminarily compare mean differences in key constructs among the three teacher groups and to compute the Pearson correlation matrix for all variables. In the second stage, Mplus 8.3 was used to perform confirmatory factor analysis (CFA), measurement invariance testing, and multi group structural equation modeling (SEM).

The measurement model was estimated using the maximum likelihood method. Model fit was evaluated using the following indices and their corresponding criteria (Hu and Bentler, 1999): a chi square to degrees of freedom ratio (*χ*^2^/df) of less than 3 indicated good fit, and less than 5 indicated acceptable fit; comparative fit index (CFI) and Tucker Lewis index (TLI) values of 0.90 or higher indicated acceptable fit, and 0.95 or higher indicated good fit; root mean square error of approximation (RMSEA) values of less than 0.08 indicated acceptable fit, and less than 0.06 indicated good fit; standardized root mean square residual (SRMR) values of less than 0.08 indicated acceptable fit.

Convergent validity was assessed using composite reliability (CR > 0.70) and average variance extracted (AVE > 0.50). Discriminant validity was examined using the Fornell Larcker criterion, requiring that the square root of the AVE for each construct be greater than its correlations with other constructs, as well as the heterotrait monotrait (HTMT) criterion, requiring HTMT values below 0.85 (Schermelleh-Engel et al., 2003; Henseler et al., 2015).

Common method bias was tested using two approaches. First, Harman’s single factor test was conducted by performing an unrotated exploratory factor analysis on all measurement items. The results showed that the first factor explained 28.6% of the total variance, which is below the recommended threshold of 40%, indicating that common method bias was not a serious concern (Podsakoff et al., 2003). Second, the unmeasured latent method factor approach was employed by adding a common method factor to the CFA model, allowing all items to load simultaneously on their respective constructs and on the method factor. After introducing the method factor, the model fit did not improve significantly (ΔCFI = 0.002, ΔRMSEA = 0.001), and the factor loadings of all items on their original constructs remained significant (*p* < 0.001), further confirming that common method bias did not affect the substantive conclusions.

Although Harman’s single-factor test and the unmeasured latent method factor approach are commonly used to assess common method bias, both have been criticized for their limited sensitivity. We acknowledge that these statistical remedies do not substitute for design-based procedural controls, such as temporal separation of predictor and criterion variable measurement or multi-source data collection. Given the cross-sectional nature of our survey design, these procedural remedies were not feasible in the current study. Future research should consider incorporating such design-based remedies to further strengthen causal inferences and reduce the risk of common method bias.

### 3.4 Measurement invariance testing

The validity of multi group comparisons presupposes that measurement invariance holds across different regional groups. This study employed multi group confirmatory factor analysis (MGCFA) to test three levels of invariance sequentially: configural invariance, metric invariance, and scalar invariance.

Configural invariance requires that eastern, central, and western groups share the same factor structure. The freely estimated model yielded the following fit indices: *χ*^2^/df = 2.84, CFI = 0.94, TLI = 0.93, RMSEA = 0.06, SRMR = 0.05, indicating that configural invariance was established.

Metric invariance was tested by constraining the factor loadings to be equal across the three groups based on the configural invariance model. Compared with the configural invariance model, the metric invariance model showed the following changes in fit: ΔCFI = −0.003, ΔRMSEA = +0.001, ΔSRMR = +0.002. All changes were within the recommended criterion of ΔCFI ≤ 0.01 proposed by Cheung and Rensvold (2002), indicating that metric invariance was established. This means that the measurement items for each construct had the same meaning across the three regional groups of teachers, making cross regional path coefficient comparisons valid.

Scalar invariance was tested by further constraining the measurement intercepts to be equal across groups based on the metric invariance model. The fit differences between the metric invariance model and the scalar invariance model were ΔCFI = −0.004 and ΔRMSEA = +0.002. Although the chi square difference test for scalar invariance reached statistical significance (Δ*χ*^2^ = 48.27, Δdf = 24, *p* < 0.01), the ΔCFI value remained below the 0.01 threshold. According to Chen’s recommendations (Cheung and Rensvold, 2002), scalar invariance can be considered as approximately established when ΔCFI ≤ 0.01 and ΔRMSEA ≤ 0.015.

It should be noted that the primary focus of this study is on comparing path coefficients across groups rather than comparing latent means. According to Kline (2023), path coefficient comparisons require only metric invariance, and scalar invariance is not a necessary condition. Therefore, the establishment of metric invariance already provides a sufficient statistical basis for multi group path analysis.

To further confirm the robustness of measurement invariance, this study conducted a sensitivity analysis using the alignment method (Asparouhov and Muthén, 2014). This approach does not require strict scalar invariance but instead assesses cross group comparability by minimizing parameter non-invariance. The results showed that the proportion of non-invariant parameters across the three groups was less than 15%, with CFI = 0.93 and RMSEA = 0.06, further supporting the reliability of cross group comparisons.

## 4 Results

### 4.1 Descriptive statistics and correlation analysis

Table 2 presents the means, standard deviations, and Pearson correlation coefficients for all study variables. From a regional perspective, eastern teachers had significantly higher mean scores than their central and western counterparts on performance expectancy (*M* = 5.67, SD = 0.92), social influence (*M* = 5.48, SD = 0.96), and adoption intention (*M* = 5.72, SD = 0.89) (*p* < 0.01). In contrast, western teachers had significantly lower mean scores than eastern teachers on effort expectancy (*M* = 4.89, SD = 1.08) and facilitating conditions (*M* = 4.76, SD = 1.12) (*p* < 0.001). This pattern provides preliminary support for the presence of structural differences in digital teaching perceptions and intentions across regions.

**Table 2:** Descriptive statistics and correlation matrix (full sample).

Variable	*M*	SD	1	2	3	4	5	6	7	8	9
1. Gender	1.53	0.50	–								
2. Age	38.6	9.20	−0.04	–							
3. Teaching experience	12.8	8.50	−0.06	0.92**	–						
4. Professional title	2.62	0.89	−0.02	0.68**	0.71**	–					
5. Performance expectancy	5.42	1.01	0.03	0.12*	0.11*	0.14**	(0.85)				
6. Effort expectancy	5.08	1.12	−0.01	0.08	0.09	0.10*	0.45**	(0.82)			
7. Social influence	5.21	1.05	0.05	0.06	0.07	0.09*	0.51**	0.39**	(0.79)		
8. Facilitating conditions	5.03	1.18	−0.02	0.10*	0.09*	0.13**	0.48**	0.56**	0.42**	(0.84)	
9. Adoption intention	5.48	1.06	0.04	0.13*	0.12*	0.15**	0.58**	0.41**	0.49**	0.52**	(0.89)

*N* = 351. M = mean. SD = standard deviation. Gender: 1 = male, 2 = female. Professional title: 1 = assistant professor, 2 = lecturer, 3 = associate professor, 4 = full professor. Values in parentheses on the diagonal are the square roots of the average variance extracted (AVE). *p < 0.05, **p < 0.01 (two tailed).

The correlation analysis at the full sample level revealed that performance expectancy was significantly and positively correlated with adoption intention (*r* = 0.58, *p* < 0.01). Similarly, effort expectancy (*r* = 0.41, *p* < 0.01), social influence (*r* = 0.49, *p* < 0.01), and facilitating conditions (*r* = 0.52, *p* < 0.01) all showed significant positive correlations with adoption intention. All correlations between the independent variables and the dependent variable were moderate to strong in magnitude and aligned with theoretical expectations, providing initial support for subsequent hypothesis testing. Furthermore, all correlations among latent variables were below 0.70, and the square roots of the AVEs, ranging from 0.79 to 0.89, were all greater than the correlations between each construct and other constructs, indicating good discriminant validity.

### 4.2 Measurement model testing

Before testing the structural model, confirmatory factor analysis (CFA) was conducted to assess the factor structure, convergent validity, and discriminant validity of the latent variables. The full sample five factor model, comprising performance expectancy, effort expectancy, social influence, facilitating conditions, and adoption intention, yielded the following fit indices: *χ*^2^/df = 2.96 (*χ*^2^ = 398.47, df = 135), CFI = 0.95, TLI = 0.94, RMSEA = 0.06 (90% CI = [0.05, 0.07]), and SRMR = 0.04. All fit indices met or exceeded the recommended standards, indicating that the five factor model fit the data well (Hu and Bentler, 1999).

Convergent validity was assessed through factor loadings, composite reliability (CR), and average variance extracted (AVE). As shown in Table 1, the standardized factor loadings for all measurement items ranged from 0.77 to 0.90 (*p* < 0.001), all exceeding the recommended threshold of 0.70. Composite reliability for each latent variable ranged from 0.87 to 0.92, exceeding the 0.70 criterion, and AVE values ranged from 0.63 to 0.80, exceeding the 0.50 criterion (Fornell and Larcker, 1981). These results collectively indicate that the measurement model had good convergent validity.

Discriminant validity was examined using two approaches. First, the Fornell-Larcker criterion, as shown in Table 2, indicated that the square roots of the AVEs for all latent variables, ranging from 0.79 to 0.89, were all greater than the correlations between each construct and other constructs, with the highest correlation being 0.58. Second, the heterotrait monotrait (HTMT) criterion results showed that all HTMT values among construct pairs were below the conservative threshold of 0.85, with the highest value being 0.73. Both methods consistently demonstrated that the five latent variables in this study had good discriminant validity.

To further confirm the equivalence of the measurement structure across the three groups, separate CFAs were conducted for the eastern, central, and western subsamples. The results showed that the CFI values for the three groups were 0.94, 0.93, and 0.94, respectively, and the RMSEA values were 0.07, 0.07, and 0.06, respectively. All values fell within the acceptable range, indicating that the measurement structure was stable across different regional samples.

### 4.3 Full sample structural model

For the full sample without distinguishing regions, a UTAUT core path structural model was constructed to test H1 through H4. The model fit the data well: *χ*^2^/df = 3.24 (*χ*^2^ = 486.71, df = 150), CFI = 0.93, TLI = 0.92, RMSEA = 0.07 (90% CI = [0.06, 0.08]), and SRMR = 0.05.

The structural path analysis results are presented in Figure 1. Performance expectancy had a significant positive effect on adoption intention (*β* = 0.32, *p* < 0.001), supporting H1. Effort expectancy had a significant positive effect on adoption intention (*β* = 0.14, *p* < 0.01), supporting H2. Social influence had a significant positive effect on adoption intention (*β* = 0.21, *p* < 0.001), supporting H3. Facilitating conditions had a significant positive effect on adoption intention (*β* = 0.26, *p* < 0.001), supporting H4. The four independent variables jointly explained 51.2% of the variance in adoption intention (*R*^2^ = 0.51), indicating that the model had moderate to strong explanatory power.

Regarding control variables, professional title had a marginally significant positive effect on adoption intention (*β* = 0.08, *p* < 0.05), while gender, age, teaching experience, and discipline category did not reach statistical significance (*p* > 0.05). This result suggests that the variance in university teachers’ digital teaching adoption intention is primarily explained by the core UTAUT constructs rather than by basic demographic characteristics.

### 4.4 Multi-group path comparison

Given that metric invariance was established, this study employed multi-group structural equation modeling to compare path coefficients across the three teacher groups from eastern, central, and western China. The analysis proceeded in two steps. First, an unconstrained model (M1) was estimated, allowing all path coefficients to vary freely across the three groups. Second, each path coefficient of interest was constrained to be equal across groups, and chi-square difference tests were used to determine whether the constraints significantly increased model misfit.

#### 4.4.1 Model fit and path coefficient descriptions

**Figure 2:**
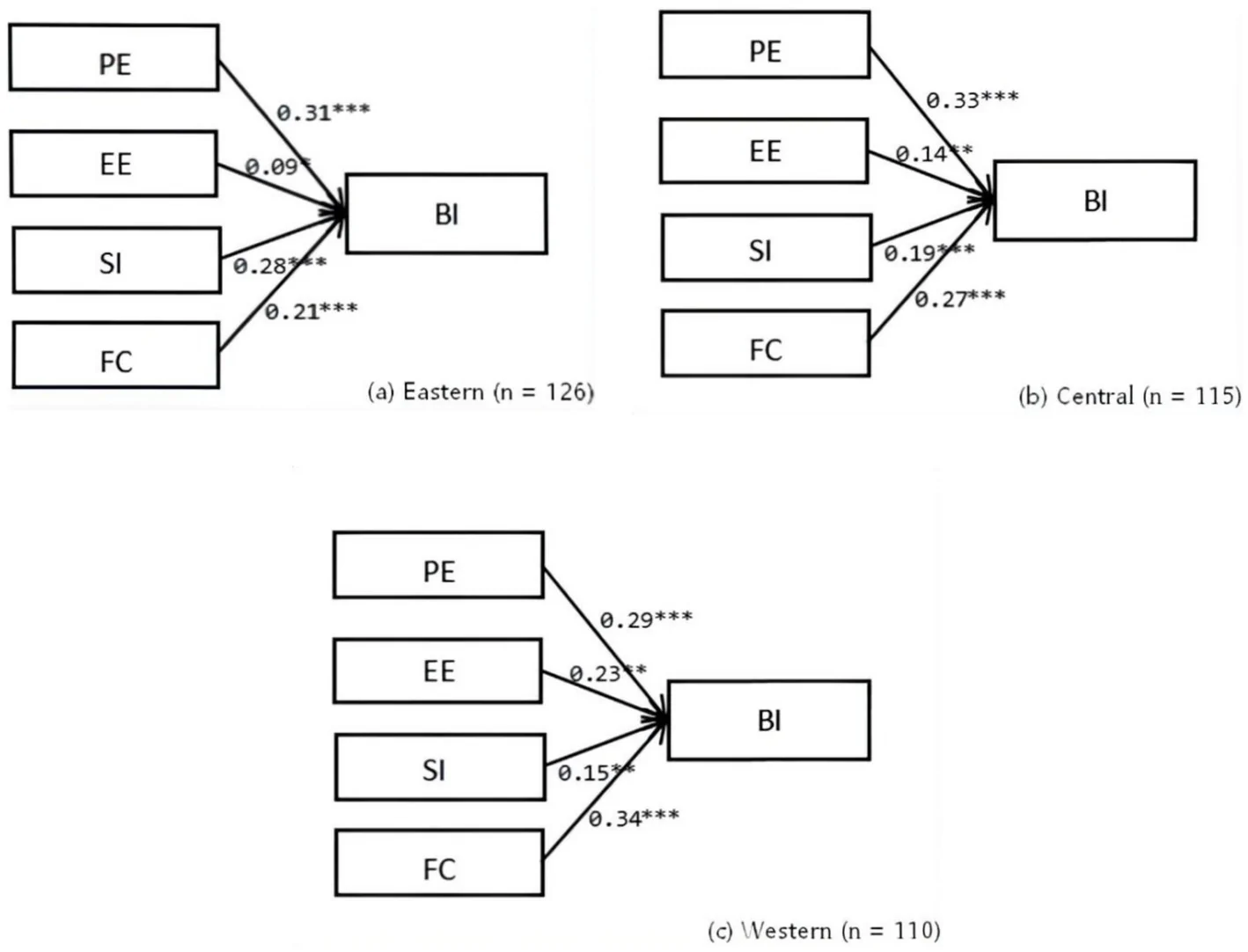
Multi-group comparison across eastern, central, and western China. **(a)** Eastern region (*n* = 126); **(b)** Central region (*n* = 115); and **(c)** Western region (*n* = 110). * indicates *p* < 0.05; ** indicates *p* < 0.01; *** indicates *p* < 0.001. Non-significant paths are shown without asterisks.

The unconstrained model (M1) yielded the following fit indices: *χ*^2^/df = 2.58 (*χ*^2^ = 1254.33, df = 486), CFI = 0.92, TLI = 0.91, RMSEA = 0.07, SRMR = 0.06. This model served as the baseline for subsequent comparisons. The structural path coefficients for the three groups are summarized in Table 3 and Figure 2.

**Table 3:** Comparison of structural path coefficients across Eastern, Central, and Western Regions.

Path	Eastern (*n* = 126)	Central (*n* = 115)	Western (*n* = 110)	Δ*χ*^2^	Δdf	*p*	Group difference pattern
	*β* (SE)	*β* (SE)	*β* (SE)				
PE → BI	0.31 (0.04)***	0.33 (0.05)***	0.29 (0.05)***	0.85	2	0.654	No significant difference
EE → BI	0.09 (0.04)*	0.14 (0.05)**	0.23 (0.06)***	9.42	2	0.009	Western > Eastern
SI → BI	0.28 (0.04)***	0.19 (0.05)***	0.15 (0.05)**	8.73	2	0.013	Eastern > Central, Eastern > Western
FC → BI	0.21 (0.05)***	0.27 (0.05)***	0.34 (0.06)***	8.16	2	0.017	Western > Eastern

β = standardized path coefficient. SE = standard error. PE = Performance Expectancy; EE = Effort Expectancy; SI = Social Influence; FC = Facilitating Conditions; BI = Adoption Intention. Δχ^2^ and Δdf are from chi-square difference tests comparing the unconstrained model with models constraining each path to be equal across groups. *p < 0.05, **p < 0.01, ***p < 0.001.

#### 4.4.2 Regional differences in the performance expectancy path (H5)

Constraining the path coefficient from performance expectancy to adoption intention to be equal across the three groups did not significantly worsen model fit compared with M1: Δ*χ*^2^ = 0.85, Δdf = 2, *p* = 0.654, ΔCFI < 0.001. This result indicates that the strength of the effect of performance expectancy on adoption intention did not differ significantly among eastern, central, and western teachers. Therefore, H5 was not supported. Descriptively, the path coefficients for the three groups were 0.31, 0.33, and 0.29, respectively. Although the western coefficient was slightly lower, the difference did not reach statistical significance.

#### 4.4.3 Regional differences in the effort expectancy path (H6)

Constraining the path coefficient from effort expectancy to adoption intention to be equal across the three groups resulted in a significant deterioration in model fit: Δ*χ*^2^ = 9.42, Δdf = 2, *p* = 0.009, ΔCFI = −0.004. Pairwise comparisons revealed that the path coefficient for western teachers (*β* = 0.23) was significantly higher than that for eastern teachers (*β* = 0.09), with a difference of 0.14 (95% CI [0.04, 0.24], *z* = 2.86, *p* = 0.004, Cohen’s *d* = 0.38). The coefficient for central teachers (*β* = 0.14) did not differ significantly from either eastern or western teachers. This result supports H6, indicating that effort expectancy has stronger explanatory power for adoption intention among western teachers than among eastern teachers.

#### 4.4.4 Regional differences in the social influence path (H7)

Constraining the path coefficient from social influence to adoption intention to be equal across the three groups resulted in a significant deterioration in model fit: Δ*χ*^2^ = 8.73, Δdf = 2, *p* = 0.013, ΔCFI = −0.004. Pairwise comparisons showed that the path coefficient for eastern teachers (*β* = 0.28) was significantly higher than that for central teachers (*β* = 0.19, difference = 0.09, 95% CI [0.01, 0.17], *z* = 2.21, *p* = 0.027, Cohen’s *d* = 0.36) and western teachers (*β* = 0.15, difference = 0.13, 95% CI [0.04, 0.22], z = 2.98, *p* = 0.003, Cohen’s *d* = 0.42). The difference between central and western teachers was not significant (*z* = 0.92, *p* = 0.358). This result supports H7, indicating that social influence has stronger explanatory power for adoption intention among eastern teachers than among central and western teachers.

#### 4.4.5 Regional differences in the facilitating conditions path (H8)

Constraining the path coefficient from facilitating conditions to adoption intention to be equal across the three groups resulted in a significant deterioration in model fit: Δ*χ*^2^ = 8.16, Δdf = 2, *p* = 0.017, ΔCFI = −0.004. Pairwise comparisons showed that the path coefficient for western teachers (*β* = 0.34) was significantly higher than that for eastern teachers (*β* = 0.21), with a difference of 0.13 (95% CI [0.03, 0.23], *z* = 2.62, *p* = 0.009, Cohen’s *d* = 0.35). The coefficient for central teachers (*β* = 0.27) did not differ significantly from that of eastern teachers (*z* = 1.33, *p* = 0.184) or western teachers (*z* = 1.48, *p* = 0.139). This result supports H8, indicating that facilitating conditions have stronger explanatory power for adoption intention among western teachers than among eastern teachers.

#### 4.4.6 Synthesis of multi-group comparison results

Taken together, three of the four core paths exhibited significant regional differences, and the pattern of differences showed clear gradients. For effort expectancy and facilitating conditions, the effect strengths were significantly higher for western teachers than for eastern teachers. For social influence, the effect strength was significantly higher for eastern teachers than for central and western teachers. Performance expectancy remained stable across the three groups. This pattern suggests that region, as a structural variable, does not uniformly moderate all influence paths but rather selectively affects specific mechanisms. Specifically, internally oriented support paths, namely effort expectancy and facilitating conditions, are more critical in resource scarce western regions, whereas externally oriented normative paths, namely social influence, are more critical in professionally dense eastern regions.

Given that multiple pairwise comparisons were conducted, the Bonferroni correction was applied to control the familywise error rate, setting the adjusted significance threshold at *p* < 0.017. Under this criterion, the western greater than eastern difference for the EE path (original *p* = 0.004) and the eastern greater than western difference for the SI path (original *p* = 0.003) remained significant. For the FC path, the western greater than eastern difference had an original *p* = 0.009, which became *p* = 0.027 after Bonferroni correction, still below 0.05 but with relatively weaker robustness, suggesting that the effect size of this difference is small and requires further validation in future research.

### 4.5 Summary

Based on the core path model of UTAUT, this study proposed eight research hypotheses and tested them through full sample structural model analysis and multi-group path comparisons. The hypothesis testing results are summarized in Table 4.

**Table 4:** Summary of hypothesis testing results.

Hypothesis	Path	Result
H1	Performance expectancy → Adoption intention	Supported
H2	Effort expectancy → Adoption intention	Supported
H3	Social influence → Adoption intention	Supported
H4	Facilitating conditions → Adoption intention	Supported
H5	Regional differences in the performance expectancy path	Not supported
H6	Effort expectancy path: Western > Eastern	Supported
H7	Social influence path: Eastern > Central and Western	Supported
H8	Facilitating conditions path: Western > Eastern	Supported

At the full sample level, performance expectancy (H1), effort expectancy (H2), social influence (H3), and facilitating conditions (H4) all had significant positive effects on digital teaching adoption intention. All four hypotheses were supported.

At the cross-regional level, three paths exhibited significant differences across regions, supporting H6, H7, and H8. Specifically, effort expectancy and facilitating conditions had stronger explanatory power for western teachers than for eastern teachers, while social influence had stronger explanatory power for eastern teachers than for central and western teachers. Although performance expectancy had the strongest effect among all predictors in the full sample, its path coefficients did not differ significantly across the eastern, central, and western groups. Thus, H5 was not supported.

In summary, seven of the eight hypotheses received empirical support, with only H5 failing to be supported.

## 5 Discussion

### 5.1 Universality of core paths: applicability of UTAUT to teachers’ digital teaching adoption

This study found that at the full sample level, performance expectancy, effort expectancy, social influence, and facilitating conditions all had significant positive effects on digital teaching adoption intention, jointly explaining 51.2% of the variance in adoption intention. This finding is highly consistent with meta-analytic conclusions from existing research on teacher technology adoption, further validating the strong explanatory power of UTAUT in the higher education context (Chen and Hwang, 2019; Buabeng-Andoh and Baah, 2020; Alowayr, 2022).

The gradient of effects across paths warrants further discussion. Performance expectancy (*β* = 0.32) and facilitating conditions (*β* = 0.26) emerged as the two strongest predictors, followed by social influence (*β* = 0.21), while effort expectancy (*β* = 0.14) showed the weakest relative contribution (García de Blanes Sebastián et al., 2025; Abdekhoda et al., 2022). This gradient pattern reflects the rational decision making characteristics of university teachers as professional practitioners (Gunasinghe et al., 2020; Alyoussef, 2021). First, performance expectancy ranked first, indicating that teachers are most concerned with whether digital teaching can bring observable pedagogical gains, such as increasing student engagement, optimizing teaching evaluation outcomes, and innovating course design formats. This aligns with the core assumption of Rational Action Theory, which posits that teachers, as bounded rational actors, allocate their time and energy to instructional activities with the highest expected returns (Alvi, 2021; Abd Rahman et al., 2021). Within this framework, performance expectancy represents teachers’ subjective utility assessment of digital teaching—a cognitive evaluation that directly drives behavioral intention. The prominence of this path across the full sample suggests that utility-based reasoning remains the primary psychological mechanism underlying teachers’ technology adoption decisions, even when contextual support and social pressure are also at play. Second, facilitating conditions ranked second. This finding highlights UTAUT’s advantage over TAM, namely that adoption behavior can be hindered by a lack of adequate hardware, training, or technical support even when individual intentions are fully present. For university teachers, the perception of facilitating conditions is not merely an operational matter of whether they can use digital teaching but also serves as a signal of whether the institution values digital teaching (Yakubu et al., 2025). Third, effort expectancy contributed the weakest effect. This finding is consistent with some meta-analytic results. A possible explanation is that university teachers generally possess high learning ability and self-efficacy. Once they overcome the initial learning threshold, ease of use no longer poses a major barrier. Furthermore, after years of development, digital teaching tools have significantly improved user experience, greatly reducing operational barriers (Wang and Nah, 2024).

From a cross regional perspective, the UTAUT core model demonstrated good fit across all three regions, eastern, central, and western, with CFI values of 0.94, 0.93, and 0.94, respectively. This indicates that the structure of the model is stable across regions. This finding provides a foundation for subsequent path comparisons, suggesting that while the model is applicable across regions, the relative weights of individual paths may differ.

### 5.2 In depth discussion of regional differences: three mechanisms and their implications

The core finding of this study lies in revealing the uneven moderating effect of region on UTAUT paths. Three of the four core paths exhibited significant regional differences. Each is discussed in depth below.

#### 5.2.1 Compensation mechanism: western strengthening of effort expectancy and facilitating conditions

This study found that effort expectancy and facilitating conditions had significantly stronger effects on adoption intention for western teachers than for eastern teachers. The path coefficient for effort expectancy increased from 0.09 in the east to 0.23 in the west, and for facilitating conditions from 0.21 to 0.34. This western strengthening effect shows a clear gradient, indicating that basic conditions have stronger explanatory power for adoption intention in relatively resource scarce regions.

How can this pattern be understood? We propose a compensation mechanism as an explanation. In eastern universities, digital teaching infrastructure is relatively well developed, teacher training systems are mature, and the frequency of technology contact in daily teaching environments is higher. In this context, most teachers have already crossed the basic technological threshold, and effort expectancy and facilitating conditions are no longer major constraints on adoption intention. Even if teachers perceive some operational difficulty or resource inadequacy, these factors do not fundamentally undermine their adoption intention. The situation is different in western universities. If teachers still face basic technological operational thresholds, such as logging into platforms, uploading resources, or using interactive tools, or if schools lack stable network environments and timely technical support, then these basic conditions become substantive constraints on adoption intention. According to the logic of diminishing marginal returns, in relatively resource scarce environments, improvements in basic conditions have larger marginal effects on behavioral intention. This compensatory pattern can be further understood through the lens of Cognitive Load Theory (Pan and He, 2024; Al-Adwan et al., 2025). When teachers have not yet automated basic digital operations—such as navigating platforms, uploading materials, or using interactive tools—these tasks impose substantial cognitive load that competes with instructional planning and pedagogical reflection. Under such conditions, effort expectancy remains a salient constraint because the cognitive resources required for technology use have not been freed for higher-order teaching considerations. In contrast, eastern teachers, who have internalized these skills through more systematic and frequent exposure, operate under reduced cognitive load, allowing ease of use to fade as a decision-making factor. Thus, the compensation mechanism observed in this study can be interpreted as the contextual manifestation of cognitive load reduction: in resource-scarce settings where skill automation has not yet occurred, effort expectancy retains its explanatory power; in resource-rich settings where automation has progressed, its marginal contribution diminishes.

This finding has important theoretical implications. First, it suggests that the foundational paths of UTAUT, namely effort expectancy and facilitating conditions, have stronger explanatory power in developing contexts. This phenomenon has been systematically underestimated in previous research that primarily used samples from developed regions. Second, it reveals a specific form of UTAUT’s contextual sensitivity, namely that not all paths vary with context, but rather specific paths, particularly those related to resources, are amplified in resource constrained contexts. Third, it provides a theoretical basis for compensatory educational technology policies, suggesting that investments in basic conditions yield higher marginal returns in resource scarce regions.

#### 5.2.2 Density mechanism: eastern strengthening of social influence

This study found that social influence had a significantly stronger effect on adoption intention for eastern teachers than for central and western teachers. The coefficient was 0.28 in the east, decreased to 0.19 in the central region, and further decreased to 0.15 in the west. This pattern runs in the opposite direction to the compensation mechanism, indicating that social influence has a stronger effect in more resource abundant regions.

We propose a density mechanism as an explanation. The operation of social influence depends on two conditions: the existence and visibility of reference others, and the relevance of the information conveyed by reference others to individual decision making. In denser professional communities, teachers’ decisions are more likely to be guided by peer behavior, a core insight of Social Learning Theory. According to this framework, observational learning requires two conditions: the presence of competent models whose behavior is visible, and the informational value of the observed outcomes. Eastern universities, characterized by denser academic communities and more frequent instructional reform activities, satisfy both conditions—teachers can readily observe peer models and assess the consequences of their digital teaching practices. The visibility of successful cases reduces uncertainty about the outcomes of adoption, while the density of professional interactions amplifies the normative weight of peer behavior. In western regions, where the visibility and density of such models are lower, the informational value of peer behavior is attenuated, weakening social influence as a driver of adoption intention. Thus, the density mechanism is not merely a descriptive label but reflects the differential availability of observational learning opportunities across regional contexts—a core proposition of Social Learning Theory applied to the organizational setting of higher education. Eastern universities have denser academic communities, higher frequencies of instructional reform activities, and teachers more easily observe peer demonstration behaviors and perceive institutional expectations. In this environment, what others do is not only visible but also informative, as peers’ successful experiences can reduce uncertainty, while peers’ wait and see attitudes may generate countervailing pressure. In central and western universities, if digital teaching has not yet formed widespread normative pressure, or if the behavior of reference others lacks informational value, such as when peers are also in a wait and see mode, the effect of social influence naturally diminishes.

An important question to consider is whether the stronger effect of social influence in the east implies that eastern teachers are more susceptible to peer pressure. The data from this study cannot directly answer this question, but reasonable speculation is possible. The mechanism of social influence can be distinguished into two types: internalization and compliance. Internalization refers to teachers adopting digital teaching because they identify with the values of their reference group. Compliance refers to teachers expressing adoption intention to meet external expectations. In eastern universities, given the higher maturity of professional communities and deeper digital teaching culture, social influence is more likely to operate through internalization. Teachers use digital teaching not because they are forced to do so but because everyone is doing it and doing so is effective. If this speculation holds, then the stronger social influence effect in eastern universities actually reflects a healthier digital teaching culture rather than stronger institutional pressure.

#### 5.2.3 Stability mechanism: cross-regional consistency of performance expectancy

Among all four paths, performance expectancy was the only one that did not exhibit significant regional differences (Δ*χ*^2^ = 0.85, *p* = 0.654). The path coefficients for eastern, central, and western regions were 0.31, 0.33, and 0.29 respectively, showing minimal descriptive differences that were not statistically significant. This null finding itself warrants in depth discussion.

Performance expectancy reflects teachers’ rational judgment of whether digital teaching can enhance teaching effectiveness. The evidence from this study suggests that regardless of whether teachers are located in eastern, central, or western regions, their logic on this core judgment is consistent. As long as teachers are convinced that digital teaching can bring pedagogical gains, their adoption intention increases accordingly. This finding carries at least three implications.

First, performance expectancy may be the most cross context robust construct in the UTAUT model. In technology acceptance research, performance expectancy, or perceived usefulness in TAM, has long been considered the most stable predictor. The findings of this study provide cross regional comparative evidence supporting this view.

Second, the cross regional stability of performance expectancy implies that interventions aimed at enhancing teachers’ digital teaching adoption intention may have good transferability across regions if they can effectively change teachers’ beliefs about whether digital teaching can enhance teaching effectiveness. This has important implications for policymakers. Rather than designing completely different intervention programs for different regions, it may be more effective to first focus on making teachers believe in the effectiveness of digital teaching through demonstration lessons, sharing empirical evidence of teaching outcomes, and disseminating successful peer cases.

Third, the stability of performance expectancy also offers methodological insights for future cross contextual comparative studies. In such studies, performance expectancy can serve as a benchmark path for examining the relative variability of other paths. If an intervention or contextual change alters the effect size of performance expectancy, it may indicate that the intervention has touched upon teachers’ cognitive restructuring of the core value of teaching effectiveness, which in itself would be an important research finding.

#### 5.2.4 Overall picture of the difference pattern

Synthesizing the discussion of the three mechanisms above, an overall picture of regional differences can be outlined. Eastern universities are characterized by high social influence and low foundational paths, meaning that social influence plays a prominent role while effort expectancy and facilitating conditions play relatively weaker roles. Western universities show the opposite pattern, characterized by high foundational paths and low social influence, meaning that effort expectancy and facilitating conditions play prominent roles while social influence plays a weaker role. Central universities fall between these two extremes, and most differences with the east and west did not reach statistical significance.

This pattern reveals an important theoretical insight. The path weights of UTAUT do not fluctuate randomly but systematically vary with regional development levels. Specifically, as regional development levels increase from west to east, the weight of social influence rises, while the weights of effort expectancy and facilitating conditions decline. This finding suggests that UTAUT may have a transitional pattern. In early stages of development, teachers focus on whether they can use digital teaching (effort expectancy) and whether they have the conditions to use it (facilitating conditions). In mature stages of development, teachers focus on how others use digital teaching (social influence) and what effects result from using it (performance expectancy), with performance expectancy remaining stable throughout. If this speculation holds, then UTAUT can be understood not only as a static model for explaining technology adoption but also as a theoretical framework for describing stages of technology adoption development.

### 5.3 Theoretical contributions

This study makes three theoretical contributions.

First, this study distinguishes between two types of contextual effects: model disconfirmation and path weighting. Previous cross contextual research on UTAUT has implicitly assumed a binary choice, that a model either applies or does not apply to a given context. The evidence provided by this study shows that the structure of UTAUT has cross regional generalizability, as model fit was acceptable across all three groups, yet path weights are context dependent. This finding shifts the theoretical focus of cross contextual comparisons from whether a model applies to which paths are strengthened or weakened in which contexts, thereby providing a new analytical framework for contextualized research on UTAUT.

Second, this study identifies compensation mechanism and density mechanism as mid range theoretical explanations for cross regional differences in UTAUT. This study elevates region from a descriptive label to an analytical concept with two theoretical dimensions, namely resource adequacy and community density. The compensation mechanism explains the strengthening effect of foundational paths, including effort expectancy and facilitating conditions, in resource scarce regions. The density mechanism explains the strengthening effect of the social influence path in professionally dense regions. The identification of these two mechanisms provides operationalizable conceptual tools for future research to examine the boundary conditions of UTAUT across different contexts, such as different countries, different institutional levels, and different academic disciplines.

Third, this study reveals the cross contextual robustness of performance expectancy as the core anchor of UTAUT. Among the four core paths, performance expectancy was the only one not moderated by region. This finding suggests that teachers’ rational judgment of whether digital teaching can enhance teaching effectiveness is universally applicable across contexts. Performance expectancy may occupy the position of a core anchor in the UTAUT model, representing the most stable and most transferable influence path. This identification not only responds to questions in meta analyses regarding whether performance expectancy is always the strongest predictor but also provides a benchmark path for future cross contextual comparative studies.

### 5.4 Practical implications

The findings of this study provide empirical evidence for differentiated strategies to promote digital teaching across universities in different regions.

For eastern universities, the focus of digital teaching promotion should shift from resource investment to culture cultivation. This study found that the marginal effects of effort expectancy and facilitating conditions are relatively limited in eastern universities. Continued increases in hardware investment or basic training may have diminishing returns in terms of enhancing teacher adoption intention. In contrast, the effect of social influence is most prominent in the east. This implies that eastern universities should pay greater attention to building professional communities. Specific strategies include cultivating demonstration cases, organizing peer observations, integrating digital teaching into departmental teaching culture, and establishing recognition and incentive systems for digital teaching.

For western universities, the foundation of digital teaching promotion remains resource assurance and skill threshold reduction. This study found that effort expectancy and facilitating conditions have significantly stronger explanatory power for western teachers than for eastern teachers. This implies that western universities should prioritize two issues. The first is lowering teachers’ technological operational thresholds by providing more user friendly platforms and more systematic entry level training. The second is improving infrastructure and the accessibility of technical support. It is worth noting that the effect of facilitating conditions became marginally significant after Bonferroni correction, suggesting that resource investment needs to be targeted. Not all types of facilitating conditions have the same marginal effects, and future research could further identify which specific support measures are most effective.

For central universities, a dual track strategy may be adopted, continuing to improve infrastructure while consciously cultivating a digital teaching culture. Central regions may be in a transitional state, lacking both the mature professional community culture of the east and facing the urgent resource constraints of the west. A differentiated strategy should attend to both ends simultaneously.

### 5.5 Limitations and future directions

This study has several limitations. First, the cross sectional data cannot strictly support causal inferences. Future research could employ longitudinal designs or quasi experimental studies. Second, the classification of regions into eastern, central, and western is relatively coarse and masks inter provincial differences. Future research could adopt finer classifications or continuous variables such as GDP per capita and internet penetration rates. Third, convenience sampling may introduce self-selection bias. Future research could incorporate objective behavioral data, such as learning management system login frequencies, to enhance the external validity of the findings. Fourth, scalar invariance was only partially supported, suggesting that latent mean comparisons should be interpreted with caution. Future research could further improve measurement equivalence using anchoring methods. Fifth, this study did not account for the nested structure of teachers within universities. Teachers were sampled from institutions varying in resource tiers (e.g., ‘Double First-Class’ universities vs. provincial institutions), a factor potentially confounded with regional classification. Although our stratified sampling strategy was designed to ensure representativeness across institutional levels, preliminary ICC calculations from our pilot data suggested that less than 5% of the variance in adoption intention resided at the institutional level, partially mitigating this concern. However, we did not statistically model institutional-level variance in the main analysis. Future research could employ multilevel structural equation modeling to disentangle individual, institutional, and regional effects, thereby clarifying whether the observed regional differences persist after controlling for institutional resource tiers.

## Data Availability

The original contributions presented in the study are included in the article/supplementary material, further inquiries can be directed to the corresponding author.
